# Incorporating self-reported health measures in risk equalization through constrained regression

**DOI:** 10.1007/s10198-019-01146-y

**Published:** 2020-01-08

**Authors:** A. A. Withagen-Koster, R. C. van Kleef, F. Eijkenaar

**Affiliations:** Erasmus School of Health Policy & Management, Erasmus University Rotterdam, Rotterdam, The Netherlands

**Keywords:** Health insurance, Risk equalization, Risk selection, Constrained regression, Survey data, I10-Health, G22-Insurance, insurance companies, actuarial studies, H51-Government expenditures and health

## Abstract

Most health insurance markets with premium-rate restrictions include a risk equalization system to compensate insurers for predictable variation in spending. Recent research has shown, however, that even the most sophisticated risk equalization systems tend to undercompensate (overcompensate) groups of people with poor (good) self-reported health, confronting insurers with incentives for risk selection. Self-reported health measures are generally considered infeasible for use as an explicit ‘risk adjuster’ in risk equalization models. This study examines an alternative way to exploit this information, namely through ‘constrained regression’ (CR). To do so, we use administrative data (*N* = 17 m) and health survey information (*N* = 380 k) from the Netherlands. We estimate five CR models and compare these models with the actual Dutch risk equalization model of 2016 which was estimated by ordinary least squares (OLS). In the CR models, the estimated coefficients are restricted, such that the under-/overcompensation for groups based on self-reported general health is reduced by 20, 40, 60, 80, or 100%. Our results show that CR can improve outcomes for groups that are *not* explicitly flagged by risk adjuster variables, but worsens outcomes for groups that *are* explicitly flagged by risk adjuster variables. Using a new standardized metric that summarizes under-/overcompensation for both types of groups, we find that the lighter constraints can lead to better outcomes than OLS.

## Introduction

Many health insurance systems are based on the model of regulated competition. Competition among health insurers helps to improve efficiency of health insurance systems and regulation helps to protect public objectives like individual affordability of health plans. One element of the regulatory framework is risk equalization, a mechanism that compensates health insurers for predictable spending variation across individuals [34, 35]. In the presence of premium-rate restrictions, as applied in (almost) all regulated health insurance markets, risk equalization mitigates incentives for risk selection.

Over the past decades, risk equalization systems have evolved from simple demographic models to sophisticated health-based models. An example of the latter is the model applied in the Netherlands, which includes risk adjusters based on an extensive series of demographic, socioeconomic, and morbidity-based variables. Even these sophisticated models, however, do not completely correct for predictable spending variation [13, 23, 30]. Van Kleef et al. [31] find that the Dutch risk equalization model of 2016 undercompensates health insurers for the group of consumers who reported a fair or (very) poor health status in the prior year and overcompensates them for the group of consumers who reported a (very) good health status in the prior year. On average, the former group (about 24% of the population) confronts health insurers with a predictable loss of around 500 euros per person per year, while the latter (about 75% of the population) confronts them with a predictable profit of around 180 euros per person per year [31].

Correlation between consumers’ (self-reported) health and their profitability to health insurers can be problematic for the functioning of health insurance markets. When the unprofitable groups in poor health value (specific features of) health plans differently than the profitable groups in good health, health insurers are confronted with incentives to design their plans in a way that these are more attractive to healthy consumers than to unhealthy consumers. For instance, health insurers might refrain from contracting high-quality care for unprofitable groups with particular chronic medical conditions [10, 11]. These actions, which we refer to as *selection via plan design*, threaten the efficiency of health plans [12, 15, 17, 25, 27, 36].

This paper seeks to mitigate incentives for selection via plan design by incorporating health survey information in the risk equalization model. However, *direct* use of self-reported health measures as a basis for risk adjusters is problematic, because the required survey information is not available for the entire population (which is typically considered a requirement for calculating individual-level risk equalization payments). Collecting this information for the entire population would usually be considered too cumbersome and costly [34].

Although self-reported health measures are not appropriate as a basis for risk adjusters, they can be used *indirectly* in risk equalization models through the method of constrained regression (CR). Conventional risk equalization models are usually estimated by means of ordinary least squares (OLS). Given a set of risk adjusters, OLS results in coefficients that minimize the sum of squared residuals. CR allows for estimating coefficients that minimize the sum of squared residuals *conditional* on a pre-specified under- or overcompensation (for instance zero) for specific groups [29]. Previous research has shown that application of CR can improve payment fit for groups not explicitly flagged by risk adjusters. At the same time, CR typically worsens payment fit for groups explicitly flagged by risk adjusters. Van Kleef et al. [29] have applied CR in the Dutch context for the risk equalization model 2015 and concluded that the improved payment fit for some groups can potentially outweigh the deteriorated payment fit for other groups.

The aim of this study is to examine and evaluate the use of health survey information in risk equalization through CR. To do so, we use administrative data and health survey information from the Netherlands. The administrative data are from 2013 and contain information on medical spending and risk adjuster variables for the entire Dutch population (*N* ≈ 17 m). These data are used to replicate the Dutch risk equalization model of 2016. Furthermore, we use health survey data from 2012 based on a large sample of the Dutch population (*N* ≈ 387 k). We estimate six models, that is, one base model estimated with OLS (i.e., the Dutch risk equalization model 2016) and five models estimated with CR.

Our empirical application comes with two methodological challenges. First, to meaningfully use health survey information as a basis for CR to improve risk equalization, this information must be representative for the population. As with most samples, this is not entirely the case for our survey sample. Prior studies have shown that this sample is somewhat healthier than the population [29, 37]. We address this by rebalancing the sample using a raking procedure [1, 18] to correct for mismatches between the sample and the population. Second, a metric is required to evaluate the outcomes of CR relative to OLS. We use a new standardized evaluation metric that summarizes under- and overcompensations for a cross tabulation of two types of groups, i.e., groups explicitly flagged by risk adjusters (for which previous research has demonstrated an *increase* in under-/overcompensation with CR compared to OLS) and groups not explicitly flagged by risk adjusters (for which previous research has shown a *decrease* in under-/overcompensation with CR compared to OLS). More specifically, we first calculate the total under-/overcompensation per group, take the absolute value of these total under-/overcompensations, and then sum these over the relevant groups.

The structure of this paper is as follows. “The Dutch health insurance market” section describes relevant aspects of the Dutch health insurance system. “Literature review” section summarizes the relevant theory and previous research on selection via plan design and CR. “Data and methods” section describes the data and methods for our empirical application and “Results” section presents the results. Finally, “Discussion” section summarizes and discusses the main findings.

## The Dutch health insurance market

The Dutch health insurance market has two main components: a basic health insurance and a supplementary health insurance. Supplementary health insurance operates on the basis of free competition and is beyond the scope of this research. The basic health insurance operates on the basis of regulated competition. Regulations implemented by the Dutch government to ensure individual affordability and accessibility of the basic health insurance, include an individual mandate to buy basic health insurance, annual open enrollment, community-rated premiums, risk equalization, and a standardized benefit package. The latter means that health plans have to cover a fixed set of benefits. Insurers are, however, free to selectively contract healthcare providers. Although this is intended to improve the efficiency of health care, health plans can also use this instrument to engage in selection via plan design, e.g., by not contracting good quality health care for specific unprofitable groups of consumers, also known as ‘quality skimping’ [32, 36].

Risk equalization mitigates incentives for selection via plan design, given premium-rate restrictions. The risk equalization model is used to calculate risk-adjusted payments to health plans, based on the characteristics of their insured population. The Dutch risk equalization model is comprised of three separate models: one for somatic health care, one for mental health care, and one model for copayments due to a mandatory deductible [32]. This research focuses on the model for somatic health care, which contains the following indirect indicators of health: age, gender, region, socioeconomic status, and source of income. In addition, the model includes the following series of more direct health indicators: pharmacy-based cost groups (PCGs), diagnosis-based cost groups (DCGs), multiple-year high cost groups (MHCGs), durable medical equipment cost groups (DMECGs), physiotherapy spending in the previous year, home care spending in the previous year, and geriatric rehabilitation care spending in the previous year [32]. In this paper, we refer to these direct health indicators as ‘morbidity-based risk adjusters’.

## Literature review

### Selection via plan design

The literature on (incentives for) selection via plan design originates from the work by Rothschild and Stiglitz [26], who were the first to show theoretically that insurers react to adverse selection incentives and try to attract good risks through insurance plans’ coverage and price. Glazer and McGuire [15] applied this to the health insurance market and further developed the ideas of Rothschild and Stiglitz [26] into a model of insurer and consumer behavior. Their model shows how profit-maximizing health insurers will engage in selection via plan design to attract good risks and deter bad ones, for example by creating networks in (dis)favor of some conditions and services. Breyer et al. [2] called this ‘indirect selection’. Furthermore, by applying the insights from Frank et al. [12], Ellis and McGuire [10] showed that health plan’s incentives to engage in selection via plan design depend on both ‘predictiveness’ and ‘predictability’ [10]. Services have predictiveness if use of these services correlates with use of other services covered by the health plan. Services are predictable when consumers can (to some extent) predict how much of those services which they will use during the contract period. When consumers take predicted use of services into account when choosing a health plan, they will be sensitive to differences in health plan design with regard to those services. Consequently, health insurers can influence the choice of consumers through health plan design [9, 17, 20, 22, 24]. McGuire et al. [24] added estimated demand elasticities to the predictiveness/predictability measures by studying incentives for selection via plan design in a market with risk adjustment, and again confirmed that health insurers have incentives to deter bad risks through health plan design, specifically people with a chronic disease [24]. Ellis et al. [11] concluded that incorporating demand elasticities across services is necessary to accurately assess incentives for selection via plan design.

Other studies have investigated the actual occurrence of selection via plan design in health insurance markets. For example, Cao and McGuire [3] investigated the services offered by HMOs relative to the fee-for-service (FFS) sector within the Medicare program by researching the correlation between HMOs’ market shares and the average expenditures in the FFS sector. Their hypothesis is as follows. If HMOs try to deter consumers who are more likely to use a service, i.e., high-risk individuals, they are expected to underprovide that service. Consequently, the HMOs will selectively enroll low-risk individuals with regard to that service. As more low-risk individuals enroll in HMOs, the average risk in the FFS sector will increase, resulting in higher average expenditures in the FFS sector. This all means that if service-level selection is present, the correlation between HMO market share and FFS average expenditures should be positive for services that the HMOs underprovide and negative for services they overprovide. Indeed, this is exactly what Cao and McGuire [3] find. Also Eggleston and Bir [8], Ellis et al. [9], and Decoralis and Gugliemo [6] found evidence of health plans engaging in selection via plan design. Carey [4, 5], Lavetti and Simon [19], Geruso et al. [14], and Han and Lavetti [17] found evidence for selection via plan design by health plans with regard to prescription drugs and Shepard [28] showed the same for hospital network design.

In summary, existing literature suggests that incentives for selection via plan design are a function of consumers’ expected spending for services covered by health plans and their (un)profitability to plans. Empirical studies have shown that insurers respond to these incentives via the design of their plans.

### Constrained regression

Conventional risk adjustment models are typically estimated with OLS, which—given a set of risk adjusters—results in coefficients that minimize the residual sum of squares. CR allows for estimating coefficients that minimize the residual sum of squares *conditional* on a constraint imposed by the researcher. An example of a constraint is that the under- or overcompensation for a certain group equals a specific amount, such as zero [29].

Previous research has shown that—compared to OLS—use of CR in risk equalization comes with a trade-off between improved compensation for groups not explicitly flagged by risk adjusters and worsened compensation for groups explicitly flagged by risk adjusters. To make a well-informed trade-off, Van Kleef et al. [29] argue that it is important to carefully define the groups that are vulnerable to risk selection. In addition, they argue that the relative importance of under-/overcompensations might vary with the size and sign (positive or negative) of the compensation. The authors find that under certain circumstances, the improvement in compensation for groups not explicitly flagged by risk adjusters can outweigh the deterioration in compensation of groups that are explicitly flagged by risk adjusters [29].

Van Kleef et al. [29] were not the first to study the use of CR in the context of risk equalization. Glazer and McGuire [16] already proposed including constraints in risk equalization to improve incentives for health plans. Starting from a model of insurer and consumer behavior, they showed that the optimal risk equalization coefficients result from CR with constraints for each of the separate services that health plans are able to distort. Layton et al. [21] have empirically implemented this approach. A key difference between the present study and the studies mentioned above is that here the information used as a basis for constraints does not come from administrative data that are available for the entire population, but from a health survey that is only available for a sample of the population.

## Data and methods

### Data

To study the effects of including health survey information in the Dutch risk equalization model through CR, we merge administrative data from 2013 with health survey data from 2012. The administrative data come from various administrative sources and contain information on individual-level medical spending and risk adjusters for all Dutch citizens with a basic health insurance in 2013 (*n* = 16.9 million). The health survey data contain information on self-reported general health as well as specific self-reported chronic conditions for 387,195 individuals who were 19 years or older on September 1, 2012 and come from Statistics Netherlands [37]. The data sets were merged using a unique anonymized individual-level identification key.

#### Rebalancing

The survey sample used for this study is somewhat overrepresented by relatively healthy individuals [29, 38]. To correct for differences in health as well as in age and socioeconomic factors between the survey sample and the population, we rebalanced the sample by means of a raking procedure which was originally developed by Deming [7]. This procedure generates individual-level weights that equalize the frequencies of key variables in the sample to those in the population [1, 18]. To see how this procedure works, imagine a sample that needs to be made representative for a population with respect to age and gender. As the joint distribution of these variables is only known in the sample, first a cross tabulation of age and gender is made for the sample (say 20 categories for age and 2 for gender which results in 20 × 2 = 40 cells). Next, for each separate row (say an age category), each entry of that row is multiplied by the ratio of the population total to the sample total for that age category, such that the row totals for the sample equal those for the population. Therefore, for each of the 20 rows/age categories, the 2 entries of gender are multiplied by the relevant ratio of the population total to the sample total. Then, this step is repeated for the columns (gender), after which the column totals will equal those in the population. The row totals (age categories), however, will no longer agree, although they are closer to the population totals than before the first iteration for the rows. This process is continued until agreement for both rows and columns is achieved [1, 18].

In addition to age and gender, our raking procedure includes all other risk adjuster classes of the risk equalization model 2016 (see Van Kleef et al. [32] for a complete list). Furthermore, the procedure also includes a proxy for whether or not someone had died in 2013 as well as 18 quantiles of mean total medical spending. The next section presents the representativeness of the sample before and after rebalancing.

#### Representativeness of survey sample

**Fig. 1:**
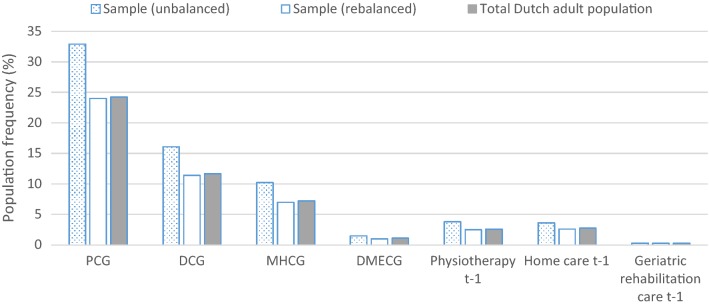
Frequencies per morbidity-based risk adjuster for the (un)balanced sample and total Dutch adult population. The horizontal axis shows groups of individuals flagged by a morbidity-based risk adjuster included in the Dutch risk equalization model 2016. The abbreviations refer to pharmacy-based cost groups (PCGs), diagnosis-based cost groups (DCGs), multiple-year high cost groups (MHCGs), and durable medical equipment cost groups (DMECG)

**Fig. 2:**
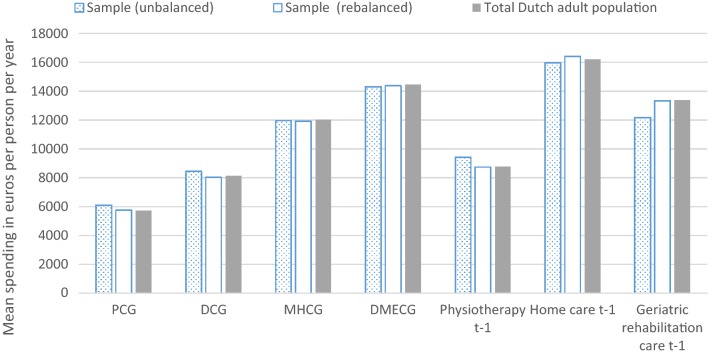
Average spending per morbidity-based risk adjuster for the (un)balanced sample and total Dutch adult population. The horizontal axis shows groups of individuals flagged by a morbidity-based risk adjuster included in the Dutch risk equalization model 2016. The abbreviations refer to pharmacy-based cost groups (PCGs), diagnosis-based cost groups (DCGs), multiple-year high cost groups (MHCGs), and durable medical equipment cost groups (DMECG)

The survey sample includes 387,195 respondents of which 384,004 successfully merged with the administrative data of 2013. Unsuccessful matches can occur due to migration and death. After removing records with missing values on self-reported general health, 379,054 individuals remained for the analyses. Figure 1 shows the relative frequency in the sample and the total population, respectively, for the seven morbidity-based risk adjusters included in the risk equalization model 2016. Before rebalancing, the sample is overrepresented by people with morbidity. After rebalancing, the relative frequencies in the sample are close to those in the total population. For the same set of risk adjusters, Fig. 2 shows the average spending in the sample and the total population, respectively. Here too, the sample matches the population relatively well, especially after rebalancing. Appendix 1 shows similar patterns for other partitions of the population.

#### Recalibrating the balanced survey sample

The Dutch risk equalization model is estimated with OLS. A property of OLS is that the mean predicted spending in the estimation data set equals the mean spending in that data set, implying that the residual spending has a mean of zero. This is not necessarily the case for subsamples drawn from the estimation data set, such as our health survey sample. Before rebalancing, the average spending in the survey sample equals 3190 euros and the predicted spending 3223 euros, leaving a mean residual of − 33 euros per person per year. After rebalancing, the mean spending equals 2429 euros and the mean predicted spending equals 2438 euros, leaving a mean residual of − 9 euros. To correct for this remaining discrepancy, we recalibrated spending in the rebalanced sample by multiplying individual-level spending by a factor of 1.003767 (= 2438/2429), so that the mean residual spending in the sample equals zero.

### Methods

#### Model specification

To analyze the effects of incorporating self-reported health measures in risk equalization via CR, we estimated six models. The first model is the actual Dutch risk equalization model of 2016 (see “The Dutch health insurance market” section) estimated with OLS. The other five models mimic the risk equalization model of 2016, but are estimated by CR. In the CR models, the under-/overcompensations of the group with a fair or (very) poor self-reported general health and the group with a (very) good self-reported general health are reduced by 20, 40, 60, 80, and 100%, respectively. Technically, imposing the constraints means that for each of the two groups of self-reported general health, the sum product of risk adjuster values and estimated coefficients (i.e., total predicted spending for a group) equals a pre-specified amount. In an initial pass, the survey data are used to determine the risk adjuster values as well as the ‘pre-specified amounts’ corresponding to the above-mentioned 20–40–60–80–100% reductions in under-/overcompensations for the relevant groups.

#### Evaluation

Prior research has shown that CR can improve compensation for some groups and worsen compensation for others. To evaluate the outcomes, we first calculated the under-/overcompensations based on the OLS model and the five CR models for selected survey groups that are *not* explicitly flagged by risk adjusters in the risk equalization model, as well as for selected groups that *are* explicitly flagged by risk adjusters in that model. Under-/overcompensation is defined as the spending predicted by the risk equalization model minus the actual spending. The survey groups are based on self-reported general health, the number of self-reported chronic conditions and specific self-reported chronic conditions (see Appendix 2). Regarding the groups explicitly flagged by risk adjusters, we focus on those flagged by the seven morbidity-based risk adjusters (see “The Dutch health insurance market” section).

Second, we constructed a standardized metric to evaluate the outcomes of CR compared to OLS in terms of group-level payment fit. Four groups are used for this part of the evaluation, i.e., yes/no (very) good self-reported general health (based on the health survey) cross tabulated with yes/no morbidity. The morbidity group is defined as being flagged by at least one of the seven morbidity-based risk adjusters of the risk equalization model and the non-morbidity group is defined as being flagged by none of the seven morbidity-based risk adjusters. In this metric, we first calculate the total under-/overcompensation for the relevant groups. Next, we take the absolute values of these total under-/overcompensations and then sum these over the groups. For simplicity and interpretation purposes, we standardize the metric by taking the ratio of the outcome for a CR model to the outcome of the OLS model. Our measure can be written as follows:

1 $$S = \frac{{\mathop \sum \nolimits_{g = 1}^{4} (\left| {\mathop \sum \nolimits_{i \in g} r_{{{\text{cr}},i}} } \right|) }}{{\mathop \sum \nolimits_{g = 1}^{4} (\left| {\mathop \sum \nolimits_{i \in g} r_{{{\text{ols}},i}} } \right|)}},$$

where: *i*ϵ*g* = the individuals belonging to group *g*; *r*_cr,*i*_ = the under-/overcompensation based on constrained regression for individual *i*; *r*_ols,*i*_ = the under-/overcompensation based on OLS for individual *i*;

When *S* > 1, the outcomes of OLS are preferred over the outcomes of CR, while the opposite holds when *S* < 1.

## Results

“Individual-level fit” section presents and compares the outcomes of the six models in terms of individual-level fit. “Mean under-/overcompensation for groups identified in the survey data” section presents the results under all six models for groups defined by self-reported general health and specific self-reported chronic conditions. The results for the groups explicitly flagged by the morbidity-based risk adjusters in the risk equalization model 2016 are presented in “Mean under-/overcompensation for groups flagged by morbidity-based risk adjusters in the risk equalization model” section. Then, “Mean per person under-/overcompensation for a cross tabulation of groups identified in the survey data and groups flagged by morbidity-based risk adjusters in the risk equalization model” section presents the outcomes of the six models in terms of metric (1). In our primary analyses, groups are weighted equally. Acknowledging that regulators might have reason to give more weight to some groups than to others, “Differentiated weighting of subgroups” section illustrates the effects of a form of differentiated weighting.

### Individual-level fit

Table 1 shows the individual-level *R*-squared^1^ and Cummings’ Prediction Measure (CPM)^2^ for each model. As can be seen, for all CR models, the *R*-squared is lower compared to that of the OLS model. Furthermore, the *R*-squared decreases as the constraint gets heavier. Under OLS, the residual sum of squares is minimized given the set of risk adjusters implying that—compared to OLS—any constraint of this type will result in a larger residual sum of squares. However, from the figures in Table 1, it can be concluded that imposing the constraints results in a very small reduction in payment fit at the individual level for both the *R*-squared and the CPM.

**Table 1:** Description and outcomes of the six models

Model	*R*-squared (%)	CPM (%)
OLS (0%)	27.9	29.9
CR-20%: constrained regression model with 20% reduction of under-/overcompensations for the two groups based on self-reported general health	27.9	30.0
CR-40%: constrained regression model with 40% reduction of under-/overcompensations for the two groups based on self-reported general health	27.8	30.0
CR-60%: constrained regression model with 60% reduction of under-/overcompensations for the two groups based on self-reported general health	27.8	29.9
CR-80%: constrained regression model with 80% reduction of under-/overcompensations for the two groups based on self-reported general health	27.7	29.7
CR-100%: constrained regression model with 100% reduction of under-/overcompensations for the two groups based on self-reported general health	27.6	29.3

### Mean under-/overcompensation for groups identified in the survey data

To illustrate the effect of imposing the constraints, Table 2 presents the mean per person under-/overcompensation based on all six models for selected survey groups. As expected, under the CR models the under-/overcompensation for the two groups defined by self-reported general health (i.e., the groups on which the constraints are based) are reduced by 20, 40, 60, 80, and 100% compared to the OLS model.^3^

**Table 2:** Mean under-/overcompensation by six models in euros per person per year for groups identified in the health survey

Survey group	Size of group (%)	Mean spending in euros (2013)	Mean under-/overcompensation per person per year in euros (2013)					
			OLS (0%)	CR-20%	CR-40%	CR-60%	CR-80%	CR-100%
Self-reported general health								
Fair, poor or very poor	27.6	5602	− 494*	− 396*	− 297*	− 198*	− 100*	− 1
Good or very good	72.4	1439	156*	125*	93*	62*	31*	− 1
Self-reported chronic condition (past 12 months)								
At least one	60.1	3376	− 122*	− 92*	− 62*	− 32*	− 2	28*
None	28.2	1010	178*	130*	83*	35*	− 13*	− 60*
1	25.6	2182	50*	41*	31*	22*	13	4
2	15.1	3095	− 126*	− 105*	− 83*	− 61*	− 39*	− 17
3	8.4	4352	− 348*	− 289*	− 229*	− 169*	− 109*	− 49*
4	11.0	6443	− 427*	− 297*	− 166*	− 36*	95*	226*
Missing	11.7	2396	26*	29*	33*	37*	41*	45*
Diabetes (ever)								
Yes	8.2	6739	− 192*	− 46*	99*	246*	392*	538*
No	86.9	2116	16	4	− 7	− 18	− 28*	− 39*
Missing	5.0	3077	− 47*	− 27*	− 6	14	34*	55*
Stroke (ever)								
Yes	4.2	7626	− 811*	− 686*	− 561*	− 435*	− 310*	− 184*
No	91.6	2251	28*	23*	18	14	9	4
Missing	4.2	2903	− 57*	− 42*	− 27*	− 12	3	18
Heart attack (ever)								
Yes	5.0	7631	− 456*	− 320*	− 184*	− 47*	89*	225*
No	90.9	2241	19	13	8	3	− 3	− 8
Missing	4.1	2955	− 76*	− 61*	− 45*	− 29*	− 14	2
Cancer (ever)								
Yes	9.9	6517	− 433*	− 351*	− 270*	− 188*	− 106*	− 24
No	86.3	2122	34*	26*	20*	13	6	− 1
Missing	3.8	2739	− 33*	− 21	− 10	2	14	26*

*OLS* ordinary least squares, *CR* constrained regression

*Statistically significantly different from zero (*P* < 0.05)

The per person undercompensation for the group with at least one self-reported chronic condition in the past year changes from − 122 under OLS to − 92 euros under CR-20% to 28 euros under CR-100%. A similar pattern can be observed for most of the other groups of chronically ill individuals. The group who has ever suffered from diabetes is on average even overcompensated by 538 euros per person per year under the CR-100% model, while OLS yields an undercompensation of 192 euros for this group. For the complementary groups of healthy individuals [i.e., those without the respective chronic condition(s)], the overcompensation generated by OLS mostly changes to an undercompensation under the CR-100% model. For example, for the group who reported no chronic condition in the last 12 months, the overcompensation of 178 euros under OLS turns into an undercompensation of 60 euros under the CR-100% model. Table 2 shows that the under- and overcompensations for all groups change linearly across the different CR models.

Table 2 also reports results for groups of survey respondents for whom the relevant information is missing. As can be seen, these groups have higher mean spending than the groups without the relevant chronic condition(s). In addition, the change in compensation when moving from OLS to CR follows the same pattern as that of the chronically ill groups, indicating that the missing groups are overrepresented by relatively unhealthy individuals.

Appendix 2 shows the same results for the 19 specific chronic conditions that survey respondents reported (not) to be suffering from in the past 12 months. Again, the chronically ill groups receive more compensation under CR than under OLS, while the overcompensations for the healthy counterparts decrease slightly.

### Mean under-/overcompensation for groups flagged by morbidity-based risk adjusters in the risk equalization model

Table 3 presents the mean per person under-/overcompensation under all six models for yes/no morbidity as well as separately for the seven morbidity-based risk adjusters in the risk equalization model of 2016. The mean under-/overcompensation for all groups is zero under OLS, except for the PCG group. The reason for this is that the PCG classes are not mutually exclusive, while the classes within all other risk adjusters are. For all other morbidity-based risk adjusters, the mean compensation under OLS is zero as this is a property of OLS. Under CR, however, this is no longer the case. As Table 3 shows, the compensation for the groups with morbidity increases as the constraint becomes heavier. Under the CR-100% model, all groups explicitly flagged by a morbidity-based risk adjuster have a mean overcompensation of at least 600 euros and the entire group of individuals flagged by at least one morbidity-based risk adjuster (yes morbidity) has a mean overcompensation of 548 euros. In contrast, the compensation for all the complementary groups of healthy people decreases. Under the CR-100% model, the entire group of individuals not flagged by a morbidity-based risk adjuster is on average undercompensated by 183 euros per person per year.

**Table 3:** Mean under-/overcompensation by six models in euros per person per year for groups (not) flagged by the morbidity-based risk adjusters of the risk equalization model 2016

Group	Size of group (%)	Mean spending in euros (2013)	Mean under-/overcompensation per person per year in euros (2013)					
			OLS (0%)	CR-20%	CR-40%	CR-60%	CR-80%	CR-100%
Morbidity								
Yes	25.0	5584	2	111*	220*	330*	439*	548*
No	75.0	978	− 1	− 37*	− 74*	− 110*	− 147*	− 183*
PCG								
Yes	19.3	5669	15*	134*	255*	375*	496*	616*
No	80.7	1286	− 3*	− 32*	− 61*	− 90*	− 118*	− 147*
DCG								
Yes	9.3	8179	0	145*	291*	437*	583*	729*
No	90.7	1514	0	− 15*	− 30*	− 45*	− 59*	− 74*
MYHCG								
Yes	5.8	12,137	0	211*	423*	634*	846*	1057*
No	94.2	1524	0	− 13*	− 26*	− 38*	− 51*	− 64*
DMECG								
Yes	0.9	14,727	0	167*	335*	502*	670*	838*
No	99.1	2020	0	− 1	− 3	− 4*	− 6*	− 7*
Physiotherapy *t*-1								
Yes	2.0	8769	0	156*	313*	470*	627*	784*
No	98.0	1998	0	− 3*	− 6*	− 9*	− 13*	− 16*
Home care *t*-1								
Yes	2.2	16,658	0	231*	463*	695*	927*	1158*
No	97.8	1827	0	− 5*	− 10*	− 15*	− 19*	− 24*
Geriatric rehabilitation care *t*-1								
Yes	0.2	13,372	0	210*	422*	633*	844*	1055*
No	99.8	2109	0	0	− 1	− 1	− 2	− 2

Morbidity is defined as being classified in one of the seven morbidity-based risk adjusters of the risk equalization model. No morbidity is defined as being classified in none of the seven morbidity-based risk adjusters of the risk equalization model 2016

*OLS* ordinary least squares, *CR* constrained regression, *PCGs* pharmacy-based cost groups, *DCGs* diagnosis-based cost groups, *MHCGs* multiple-year high cost groups, *DMECG* durable medical equipment cost groups

*Statistically significantly different from zero (*P* < 0.05)

### Mean per person under-/overcompensation for a cross tabulation of groups identified in the survey data and groups flagged by morbidity-based risk adjusters in the risk equalization model

**Fig. 3:**
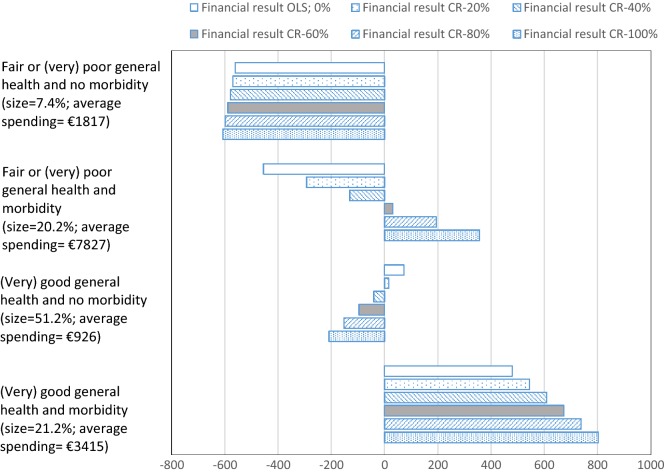
Mean under-/overcompensations under six models in euros per person per year for groups based on a cross tabulation of self-reported general health by yes/no morbidity. The abbreviations stand for ordinary least squares (OLS) and constrained regression (CR). Morbidity is defined as being classified in one of the seven morbidity-based risk adjusters of the risk equalization model. No morbidity is defined as being classified in none of the seven morbidity-based risk adjusters of the risk equalization model

Even though average compensation increases for chronically ill groups, this is not necessarily the case for subsamples of these groups. To illustrate this, Fig. 3 cross tabulates the two groups based on self-reported general health and the two groups based on yes/no morbidity as identified by the risk equalization model. The results show that within the group with a fair or (very) poor self-reported general health, the individuals flagged by a morbidity indicator (20.2% of population) receive more compensation under CR than under OLS (i.e., from − 455 euros under the OLS (0%) to 356 euros per person per year under the CR-100% model). However, the group of individuals who reported their health to be fair or (very) poor but who are not flagged by a morbidity indicator (7.4% of population), receive slightly less compensation under CR. As a result, the compensation for this subgroup decreases from − 564 euros under OLS (0%) to − 608 euros per person per year under the CR-100% model. A similar pattern can be observed within the group of people who reported a (very) good general health. The individuals who reported a (very) good general health and who are flagged by a morbidity indicator in the risk equalization model (21.2% of population) receive more compensation under CR than under OLS (i.e., from 479 euros under OLS (0%) to 802 euros per person per year under the CR-100% model). The individuals who reported a (very) good general health but are not flagged by a morbidity indicator (51.2% of population) receive less compensation under CR than under OLS (i.e., from 72 euros under OLS (0%) to − 209 euros per person per year under the CR-100% model).

**Fig. 4:**
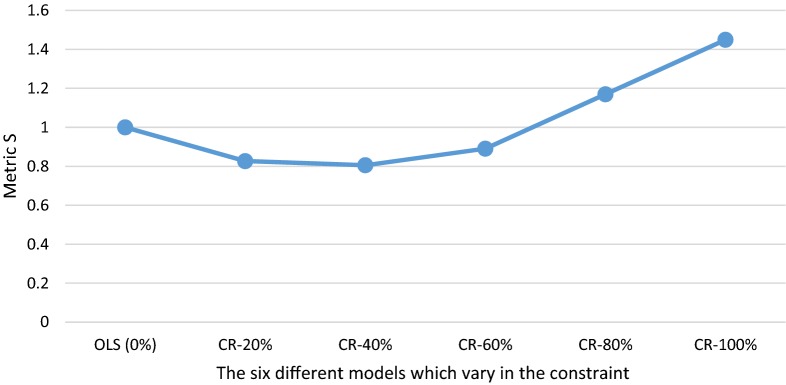
Outcomes of six models for metric S for groups based on a cross tabulation of self-reported general health and yes/no morbidity. The abbreviations stand for ordinary least squares (OLS) and constrained regression (CR). Metric *S* is calculated using Eq. (1). The constraint is a % reduction in under- and overcompensation on the group with a (very) good general health and the group with a fair or (very) poor general health

In Fig. 3, it is not obvious which of the models leads to the best outcomes overall. Figure 4 presents the outcomes of metric *S* (Eq. 1) which summarizes the outcomes over the four subgroups presented in Fig. 3. This metric first calculates the total under-/overcompensation per group, takes the absolute value of these total under-/overcompensations and sums these over the four groups. The metric compares the outcomes of a CR model relative to OLS. When *S* < 1, a CR model outperforms OLS, while *S* > 1 implies the opposite. Figure 4 shows that *S* < 1 for the CR-20%, CR-40%, and CR-60% models, indicating that these models perform better than OLS with respect to the groups analyzed here. The CR-40% model has the lowest *S*-value (i.e. 0.81), indicating that overall this model performs best (given our choice of groups). For the CR-80% and CR-100% models, *S* > 1, indicating that these models perform worse than OLS (0%), with respect to these groups. In addition, we also see that CR in risk equalization can be pushed too far: applying a stricter constraint can cause the S-value to increase again.

### Differentiated weighting of subgroups

In Fig. 4 (the under-/overcompensations of), all four groups are weighted equally. In practice, however, regulators might have reason to give more weight to some groups than to others. This might be the case when the regulator believes that some selection actions are more harmful than others. “Literature review” section supports this as it shows that under-/overcompensation and the resulting selection actions for some groups might be more problematic compared to others. For example, the regulator might consider ‘quality skimping’ through selection via plan design to be more harmful for the functioning of the healthcare system than ‘selective marketing’. In such a situation, the regulator might give more weight to groups that are particularly vulnerable to ‘quality skimping’ (e.g., groups of chronically ill people with high expected spending) than to groups that are more likely to be subject to ‘selective marketing’ (e.g., groups of healthy people). In addition, the regulator might consider an undercompensation, which incentivizes insurers to underserve people, to be more harmful than an overcompensation, which incentivizes insurers to overserve people. Although it is not our goal here to advocate a specific form of differentiated weighting of subgroups, we believe that it is instructive to indicate how weighting could influence the outcomes of the models simulated here.

**Fig. 5:**
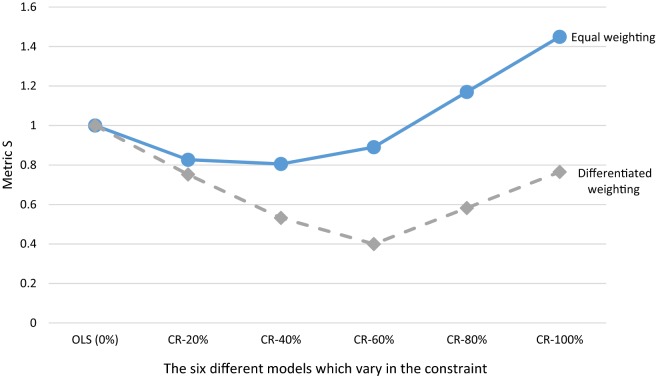
Outcomes of six models for metric S with equal weighting and differentiated weighting of subgroups. The outcomes with equal weighting of subgroups are calculated using Eq. (1). Differentiated weighting means that the result for each of the four groups is weighted with the average spending of that group and that an undercompensation is weighted twice as heavy as an overcompensation. The horizontal axis represents the different models. The series ’equal weighting’ is equivalent to the outcomes of Fig. 4

Figure 5 compares the results of the CR models under equal weighting of subgroups with those under differentiated weighting of subgroups. The data series ‘equal-weighting’ is equivalent to the results of Fig. 4. The data series ‘differentiated weighting’ presents the results of the CR models relative to OLS with two types of differentiated weighting: (1) groups with high expected spending are given more weight than those with low expected spending (in our illustration: through weighting with the average spending of the groups) and (2) undercompensations are given more weight than overcompensations (in our illustration: through weighting an undercompensation twice as heavy as an overcompensation). A regulator might consider the first type of weighting when it is particularly concerned about quality skimping, for instance through selection via plan design. A regulator might think about the second type of weighting when ‘underserving’ is considered more harmful than ‘overserving’.

Figure 5 shows that the line for ‘differentiated weighting’ of subgroups lies below the line of ‘equal-weighting’ of subgroups. This indicates that differentiated weighting of subgroups can substantially affect the outcomes of constrained regression compared to OLS. The results in Fig. 5 also show that under ‘differentiated weighting’, the lowest value for *S* is to be found for the CR-60% model instead of the CR-40% model. This indicates that the optimal specification of a constraint can be affected by how a regulator weights the subgroups of interest.

## Discussion

Most health insurance markets with premium-rate restrictions include a risk equalization system to compensate health insurers for predictable variation in spending. Recent research has shown, however, that even the most sophisticated risk equalization systems tend to undercompensate (overcompensate) people with poor (good) self-reported health, which confronts insurers with selection incentives. Self-reported health measures are generally considered infeasible for use as ‘risk adjusters’ in the risk equalization model. The aim of this paper was to examine and evaluate an alternative way of including self-reported health measures in risk equalization, namely through constrained regression (CR). To do so, we estimated five CR models and compared these with the actual Dutch risk equalization model of 2016 estimated with ordinary least squares (OLS). In the CR models, coefficients were estimated by least-squares regression given that the under-/overcompensation for two groups based on self-reported general health are reduced by 20, 40, 60, 80, or 100%.

We first calculated the under- and overcompensations for selected survey groups and groups flagged by the morbidity-based risk adjusters included in the risk equalization model. For the survey groups, the results showed that the chronically ill receive more compensation under CR compared to OLS, while the opposite is true for the complementary groups of healthy people. We observed a similar pattern for the groups (not) explicitly flagged by a morbidity-based risk adjuster; the groups that were explicitly flagged by such a risk adjuster receive more compensation under CR compared to OLS and the groups not explicitly flagged receive less. Next, we researched subsamples of these groups by cross tabulating the groups yes/no (very) good self-reported general health with the groups yes/no explicitly flagged by at least one morbidity-based risk adjuster. The results showed that—compared to OLS—also within the groups of self-reported general health, the CR models move money from the individuals not flagged by a morbidity-based risk adjuster to those flagged by such a risk adjuster. Consequently, we found that payment fit improves for some groups but worsens for others. Van Kleef et al. [33] reported similar findings.

To evaluate the outcomes under all six models, we constructed a standardized metric that summarizes the absolute under-/overcompensations for relevant subgroups. We evaluated the four groups resulting from the cross tabulation of yes/no (very) good self-reported general health with the groups yes/no explicitly flagged by at least one morbidity-based risk adjuster. In this metric, we take the absolute values of the total under-/overcompensations and sum these over the four groups. The metric then compares the outcomes of a CR model relative to OLS. We find that the CR-20%, CR-40%, and CR-60% models yield more preferable outcomes than OLS, with the CR-40% model yielding the best results (i.e., for the groups analyzed here). This finding shows that a relatively small constraint could already improve conventional risk equalization. This is in line with the conclusions drawn in the paper by Van Kleef et al. [29] and with the findings of the work by Glazer & McGuire [15, 16]. Glazer and McGuire [15, 16] argued that conventional risk equalization estimated with OLS might not be optimal and that overpaying groups flagged as ‘high risk’ and underpaying groups of ‘low risk’ could improve the outcomes of risk equalization. However, our results also show that CR in risk equalization can be pushed too far, since the metric increases sharply as the constraint becomes heavier, with the CR-80% and CR-100% models performing worse than OLS.

Our primary simulations assume equal weighting of (the under-/overcompensations of) subgroups. Acknowledging that regulators might consider the effects of some selection actions to be more harmful than others, we also examined how differentiated weighting could influence the model outcomes. We found that a specific form of weighting (based on assumptions about the effects of quality skimping and underserving versus overserving) substantially affects the outcomes of the CR models relative to OLS. These results demonstrate the relevance of carefully defining the policy objectives which regulators want to include in the evaluation.

The results of this study indicate that the use of health survey information in risk equalization through CR can be promising in reducing incentives for selection via plan design. Practical implementation of survey information in risk equalization through CR, however, needs more work. First, evaluation can be more refined, for example by evaluating the outcomes using other and more groups than analyzed here. In addition, more refined evaluation of risk equalization models could require a welfare approach that incorporates how incentives affect the behavior of insurers, how this behavior of insurers interacts with the behavior of consumers, and how this affects social welfare. Although such a welfare approach is beyond the scope of this paper, we believe that further research into this direction can help to improve the evaluation of risk equalization systems. Second, the choice of groups on which the constraints are based can differ from the groups used is this research. This choice is, however, not ours to make. The method of CR offers regulators an effective tool for protecting specific groups of interest against selection via plan design [29]. An important insight in this respect is that these groups can also be determined on subsamples of the population, as long as these subsamples are representative for the population.

## Figures and Tables

**Fig. 6:**
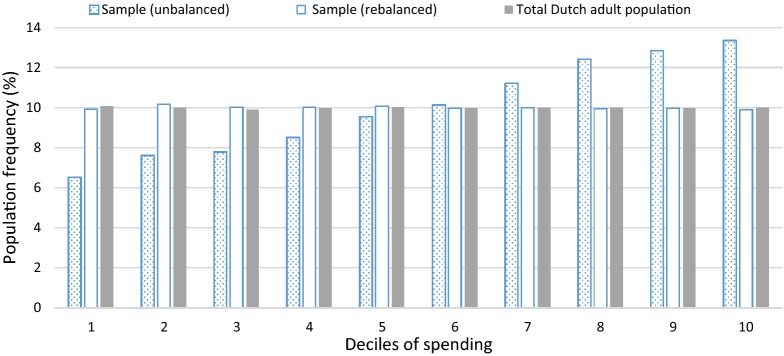
Frequencies per decile of spending for the (un)balanced sample and the total Dutch adult population. The deciles on the horizontal axis have been determined on the total Dutch adult population. The bars for the total Dutch adult population and the rebalanced sample do not exactly equal 10% due to rounding

**Fig. 7:**
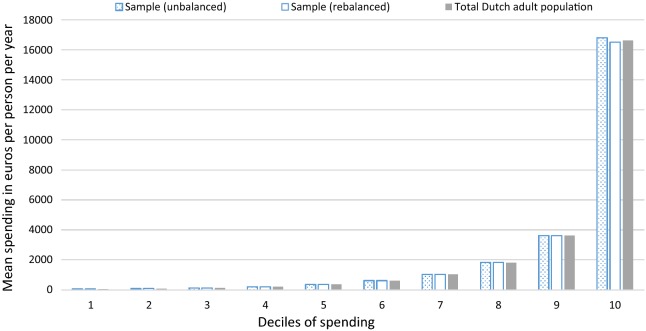
Average spending per decile of spending for the (un)balanced sample and the total Dutch adult population. The deciles on the horizontal axis have been determined on the total Dutch adult population

## Appendix 1: Representativeness of the survey sample by decile of spending

The representativeness of the sample (before and after rebalancing) for the population can also be shown using deciles of spending instead of the morbidity adjusters. Figure 6 shows the relative frequency per decile of actual medical spending for the unbalanced sample (dotted bars), rebalanced sample (empty bars), and the total adult population (solid bars). Before rebalancing, the sample is clearly overrepresented by high spenders. After rebalancing, the relative frequency per decile of spending in the survey sample matches that of the population.

Figure 7 presents the average spending per decile of spending. Patterns in the sample are in line with those in the population, before but especially after rebalancing.

## Appendix 2: Under-/overcompensation for survey groups regarding self-reported conditions under OLS and constrained regression

Table 4 shows the under-/overcompensations for survey groups regarding self-reported conditions in the last 12 months under OLS and constrained regression.

**Table 4:** Under-/overcompensations in euros for survey groups of specific self-reported conditions in the last 12 months estimated with ordinary least squares (OLS) and constrained regression (CR)

Groups	Size (%)	Mean spending in euros	Mean under-/overcompensation per person per year in euros (2013)					
			OLS (0%)	CR-20%	CR-40%	CR-60%	CR-80%	CR-100%
Stroke								
Yes	0.7	9117	− 722*	− 561*	− 399*	− 237*	− 75*	87*
No	95.3	2368	9	7	5	3	1	− 1
Missing	4.0	3074	− 92*	− 74*	− 57*	− 39*	− 21	− 4
Heart attack								
Yes	0.5	8509	− 503*	− 333*	− 162*	9	180*	351*
No	95.3	2376	7	4	3	1	− 1	− 3
Missing	4.2	3102	− 82*	− 62*	− 43*	− 23*	− 4	16
Heart condition								
Yes	3.2	8918	− 790*	− 630*	− 469*	− 309*	− 148*	12
No	92.6	2267	18	13	8	4	− 1	− 6
Missing	4.2	2908	− 7	10	27*	44*	62*	79*
Cancer								
Yes	2.8	10,444	− 1137*	− 1022*	− 905*	− 789*	− 673*	− 557*
No	93.0	2260	23*	20*	17	14	10	7
Missing	4.2	2969	− 45*	− 28*	− 11	6	23*	40*
Migraine								
Yes	12.4	2286	− 107*	− 98*	− 89*	− 81*	− 72*	− 63*
No	74.5	2341	30*	24*	17	11	5	− 1
Missing	13.1	3193	− 48*	− 23	2	28*	53*	79*
Blood pressure								
Yes	22.0	4415	− 208*	− 150*	− 91*	− 32*	27	86*
No	65.4	1897	49*	32*	15	− 2	− 19*	− 35*
Missing	12.7	3035	− 17	4	26*	48*	69*	91*
Blood vessels								
Yes	3.6	7507	− 552*	− 421*	− 291*	− 160*	− 29	102*
No	83.6	2198	20*	13	5	− 2	− 9	− 16
Missing	12.7	3081	− 28*	− 5	18	41*	64*	88*
Asthma								
Yes	8.6	4702	− 263*	− 182*	− 100*	− 19	63*	144*
No	78.9	2127	28*	16	5	− 6	− 18	− 29*
Missing	12.5	3035	− 16	6	28*	51*	73*	95*
Psoriasis								
Yes	2.9	3743	− 383*	− 349*	− 314*	− 280*	− 245*	− 211*
No	83.8	2290	13	7	2	− 3	− 8	− 13
Missing	13.3	3156	− 5	21	48*	74*	101*	127*
Eczema								
Yes	4.2	2758	− 150*	− 136*	− 122*	− 108*	− 94*	− 80*
No	83.0	2316	15	10	6	1	− 3	− 8
Missing	12.8	3142	− 44*	− 20	5	30*	54*	79*
Severe/recurring dizziness								
Yes	4.3	5772	− 502*	− 400*	− 299*	− 197*	− 95*	7
No	82.9	2180	28*	19*	11	2	− 6	− 15
Missing	12.8	3095	− 27*	− 3	21	45*	68*	92*
Severe/recurring disease of intestines								
Yes	4.4	5273	− 590*	− 514*	− 438*	− 362*	− 285*	− 209*
No	83.1	2202	34*	26*	19	12	4	− 3
Missing	12.4	3076	− 26*	− 3	20	43*	66*	90*
Incontinence								
Yes	8.2	5579	− 240*	− 154*	− 67*	19	105*	191*
No	79.0	2099	24*	13	3	− 7	− 17	− 28*
Missing	12.8	3085	− 36*	− 13	10	34*	57*	80*
Wear of joint								
Yes	18.6	4858	− 267*	− 191*	− 115*	− 39*	37*	113*
No	69.2	1929	48*	31*	14	− 2	− 19*	− 36*
Missing	12.2	2982	− 10	11	32*	53*	74*	95*
Joint inflammation								
Yes	6.4	5804	− 354*	− 241*	− 127*	− 14	100*	214*
No	80.8	2143	24*	13	3	− 8	− 18	− 28*
Missing	12.8	3076	− 18	5	29*	52*	76*	100*
Severe/recurring condition of back								
Yes	11.0	3987	− 233*	− 174*	− 115*	− 55*	4	64*
No	76.5	2151	33*	21*	10	− 1	− 12	− 23*
Missing	12.5	3023	− 20	2	25*	47*	69*	92*
Severe/recurring condition of neck								
Yes	10.1	3706	− 171*	− 117*	− 63*	− 9	45*	100*
No	77.3	2191	25*	15	5	− 5	− 15	− 25*
Missing	12.5	3064	− 32*	− 10	12	34*	56*	78*
Severe/recurring condition of elbow								
Yes	6.9	4378	− 184*	− 112*	− 39*	33*	106*	179*
No	80.4	2193	20*	10	1	− 8	− 17	− 26*
Missing	12.7	3100	− 39*	− 17	7	30*	53*	76*
Other								
Yes	15.0	4860	− 375*	− 311*	− 247*	− 183*	− 118*	− 54*
No	74.6	1900	74*	58*	43*	27*	12	− 3
Missing	10.4	3092	− 34*	− 10	14	38*	62*	86*

*Statistically significantly different from zero (*P* < 0.05)

## Footnotes

1 $$R - {\text{squared}} = 1 - \frac{{\mathop \sum \nolimits_{i = 1}^{n} \left( {y_{i} - \hat{y}_{i} } \right)^{2} }}{{\mathop \sum \nolimits_{i = 1}^{n} \left( {y_{i} - \bar{y}} \right)^{2} }}.$$

2 $${\text{CPM}} = 1 - \frac{{\mathop \sum \nolimits_{i = 1}^{n} \left| {y_{i} } \right. - \left. {\hat{y}_{i} } \right|}}{{\mathop \sum \nolimits_{i = 1}^{n} \left| {y_{i} - \left. {\bar{y}} \right|} \right.}}.$$

3 The under-/overcompensations do not exactly equal zero under the CR-100% model due to the recalibration of actual spending in the survey data (see “Recalibrating the balanced survey sample” section).
